# Statistical modeling approach for PM_10_ prediction before and during confinement by COVID-19 in South Lima, Perú

**DOI:** 10.1038/s41598-022-20904-2

**Published:** 2022-10-06

**Authors:** Rita Jaqueline Cabello-Torres, Manuel Angel Ponce Estela, Odón Sánchez-Ccoyllo, Edison Alessandro Romero-Cabello, Fausto Fernando García Ávila, Carlos Alberto Castañeda-Olivera, Lorgio Valdiviezo-Gonzales, Carlos Enrique Quispe Eulogio, Alex Rubén Huamán De La Cruz, Javier Linkolk López-Gonzales

**Affiliations:** 1 Universidad César Vallejo, Escuela de Ingeniería Ambiental, Lima, Peru; 2 Dirección General de Salud Ambiental, Lima, Peru; 3 Universidad Nacional Tecnológica de Lima Sur, Lima, Peru; 4 Universidad Nacional Agraria La Molina, Escuela de Ingeniería Ambiental, Lima, Peru; 5 Universidad de Cuenca, Facultad de Ciencias Químicas, Grupo RISKEN, Cuenca, Ecuador; 6 Universidad Tecnológica del Perú, Facultad de Ingeniería Mecánica e Industrial, Lima, Peru; 7 Facultad de Ciencias de la Salud, Universidad Peruana Los Andes, Huancayo, Peru; 8 E.P. de Ingenieria Ambiental, Universidad Nacional Intercultural de la Selva Central Juan Santos Atahualpa, La Merced, Peru; 9 Facultad de Ingeniería y Arquitectura, Universidad Peruana Unión, Lima, Peru

**Keywords:** Environmental sciences, Environmental social sciences

## Abstract

A total of 188,859 meteorological-PM$_{10}$ data validated before (2019) and during the COVID-19 pandemic (2020) were used. In order to predict PM$_{10}$ in two districts of South Lima in Peru, hourly, daily, monthly and seasonal variations of the data were analyzed. Principal Component Analysis (PCA) and linear/nonlinear modeling were applied. The results showed the highest annual average PM$_{10}$ for San Juan de Miraflores (SJM) (PM$_{10}$-SJM: 78.7 $\upmu$g/m$^{3}$) and the lowest in Santiago de Surco (SS) (PM$_{10}$-SS: 40.2 $\upmu$g/m$^{3}$). The PCA showed the influence of relative humidity (RH)-atmospheric pressure (AP)-temperature (T)/dew point (DP)-wind speed (WS)-wind direction (WD) combinations. Cool months with higher humidity and atmospheric instability decreased PM$_{10}$ values in SJM and warm months increased it, favored by thermal inversion (TI). Dust resuspension, vehicular transport and stationary sources contributed more PM$_{10}$ at peak times in the morning and evening. The Multiple linear regression (MLR) showed the best correlation (r = 0.6166), followed by the three-dimensional model LogAP-LogWD-LogPM$_{10}$ (r = 0.5753); the RMSE-MLR (12.92) exceeded that found in the 3D models (RMSE $<0.3$) and the NSE-MLR criterion (0.3804) was acceptable. PM$_{10}$ prediction was modeled using the algorithmic approach in any scenario to optimize urban management decisions in times of pandemic.

## Introduction

Air is vital and its quality is measured in the most strategic places in cities^1,2^. Particulate matter (PM) with an aerodynamic diameter $<10 \upmu \text{m}$ represents a global health problem^3^ with short or long term effects^4,5^. The fixation of this pollutant in the upper respiratory system depends on the inhalation flow rate^6^, causing cardiovascular diseases^7^ and mortality due to lung cancer^8^. Megacities are the most affected due to industrialization, energy consumption and high vehicular flow, generating social costs^9^.

The World Health Organization (WHO)^10^ has established PM$_{10}$ concentration threshold values for 24-h (50 $\upmu$g/m$^{3}$) and annual (20 $\upmu$g/m$^{3}$) average measurements, which are used as a reference in air quality monitoring programs. Meteorological monitoring also contributes to measuring the effects of meteorological variables on air quality and the environment^11^. Relative humidity (RH), temperature (T), atmospheric pressure (AP), wind speed (WS) and wind direction (WD) influence both the distribution and concentration of PM$_{10}$^12–16^. This information is used in predictive models of particulate matter to optimize the control of atmospheric emissions^17^ and to manage sustainable cities^18^.

The city of Lima in Peru, is approaching ten million inhabitants and the appearance of the COVID-19 pandemic in March 2020 led to the closure of land borders^19^ and suspension of international air transport, leading to an improvement in air quality^3,20,21^ until the start of the economic reactivation in May 2020 that activated the industrial sector and vehicular transport (approximately 22,794,136 daily trips in 2019)^22,23^.

The *Ministerio del Ambiente* (MINAM) updated the air quality standards (AQS)^24^ and established the air quality index (AQI) for PM$_{10}$ called Índice de Calidad del Aire (INCA)^25^, but there is no evidence of PM$_{10}$ prediction models in southern Lima to help improve air quality management and minimize risks to human health. The *Dirección General de Salud Ambiental* (DIGESA) of the *Ministerio de Salud* (MINSA) maintains a monitoring network for relative humidity (RH), atmospheric pressure (AP), solar radiation (SR), wind speed (WS), wind direction (WD) and PM$_{10}$ as part of the air quality health surveillance program in Lima and Callao. This network includes two stations in southern Lima: *Santiago de Surco* (SS), an urbanized and commercial sector; and *San Juan de Miraflores* (SJM), a less paved and dusty sector. Two areas of Lima that are complete opposites in terms of environmental management characteristics. In addition, SJM borders the “Panamericana Sur” highway, one of the most important roads in the country and close to cement factories. Both districts (SS and SJM) have a total of 900,000 inhabitants^26^.

In this context, statistical models are suitable to describe the complex relationship between PM$_{10}$ and meteorological variables, predicting its behavior especially in urban areas. This research applies statistical approaches to the prediction of PM$_{10}$ before and during the pandemic caused by COVID-19 in Lima. Moreover, there are a limited number of studies in Lima, which is one of the cities with the highest pollution levels in South America^27–29^. Predicting air quality with high accuracy can be problematic, but these tools are becoming increasingly important because they provide comprehensive information to prevent critical pollution episodes and reduce human exposure to this pollutants^30,31^.

The objective of this research is to contribute to the environmental management of air quality through the application of a simple and effective statistical modeling of air quality related to PM$_{10}$ concentration levels, based on meteorological parameters and with scientific support that allows authorities to optimize decision making in the control of air pollution and risks to human health. The contributions of the research are summarized below:

- Implementation of statistical modeling for time series data during 2019–2020, at two meteorological and air quality monitoring locations in southern Lima.
- The three-dimensional PM$_{10}$ forecasting model based on time series, being the first time this analysis is applied in South Lima. In addition, the principal component analysis (PCA) to evaluate the effect of meteorological variables on the behavior of PM$_{10}$.

The rest of the paper is structured as follows: “Literature review”, shows the different studies that precede this research. Then, “Materials and methods” that describes the methodology developed based on statistical modeling approaches. Also, “Results and discussion” that presents the main findings of this research compared to other studies. Finally, “Limitations” and “Conclusions” that provides the main conclusions, together with some recommendations for future research.

## Literature review

The review of studies with applications for modeling PM$_{10}$ as a function of meteorological variables did not produce information in southern Lima (SJM and SS stations), but there is some background information for other districts belonging to the city of Lima. For example, air quality was evaluated at 3 points in North Lima, using the gray grouping method^32^. In addition, the dependence of particulate matter in air on meteorological parameters was studied in the town of *Zárate* in Lima^33^. Also, PM concentrations were analyzed one month before the pandemic (February 2020) and at the beginning of the pandemic (March-April 2020)^21^. In another investigation, a multivariate repression model based on internodal correlations ranging from 0.31 to 0.49 was implemented to analyze air quality at other stations in Lima, using a low-cost sensor with IIoT technology^18^. Similarly, the Weather Research and Forecasting-Chem (WRF-Chem) model was applied to develop an operational forecast system for air quality in the Metropolitan Area of Lima and Callao (MALC)^12^. Air quality for PM$_{10}$ was also evaluated in other districts of Lima through the application of artificial neural networks (ANN), observing certain difficulties for its prediction in stations with critical pollution episodes^34^.

Applications of PM$_{10}$ models on meteorological variables are related to the use of algorithms and functional relationships^35^. In China, meteorological conditions were related to interannual PM variations through correlations using robust ANN prediction models^36^. In Iran, meteorological factors were related to the PM$_{10}$/PM$_{2.5}$ ratio using the AirQ+ model, calculating positive correlations between relative humidity (RH) and temperature (T), and negative correlations with precipitation and wind speed (WS)^37^. However, one of the most commonly used models is the multiple linear regression (MLR) model^16,38^. For example, in Bulgaria, MLR was applied relating AP-RH-T-T-PM$_{10}$, reporting moderate correlations and indicating that more factors affecting PM$_{10}$ concentration should be included^15^. In China, PM$_{10}$ and PM$_{2.5}$ were related to meteorological variables by linear and single-factor exponential regressions, and determined robust models of negative relationship for WS-PM$_{10}$ and positive relationships for T-PM$_{10}$ in warm seasons, but not in cool seasons^14^. Also, ANN and MLR models were compared for meteorological variable-PM$_{10}$ combinations without obtaining significant correlations (p >0.05) for RH^39^. Another study combined the dew point (DP)-T variables on an integral Moivre-Laplace model to predict PM$_{10}$ and PM$_{2.5}$^40^. Similarly, the T-WS-AP-RH-PM$_{10}$ factors were used, showing limitations in the single factor models, and instead applied 3D graph fits to obtain better prediction results^13^. In the present research, the methodology proved to be simple and robust and was based on the statistical modeling approach to predict PM$_{10}$ concentration and contribute to generate new tools for air quality management, aerosol control and prevention of risks associated with COVID-19.

## Materials and methods

### Area of study and dataset

Lima, the capital of Peru, has a flat morphology formed by the valleys of the *Chillón*, *Rímac* and *Lurín* rivers, and meteorological variables are influenced by the relief of the Andes mountain range, the cold Humboldt current and the South Pacific anticyclone (SPA), which generates microclimates in the city^41^ and variations in the altitudes of the TI base that influence PM$_{10}$ dispersion.

**Figure 1:**
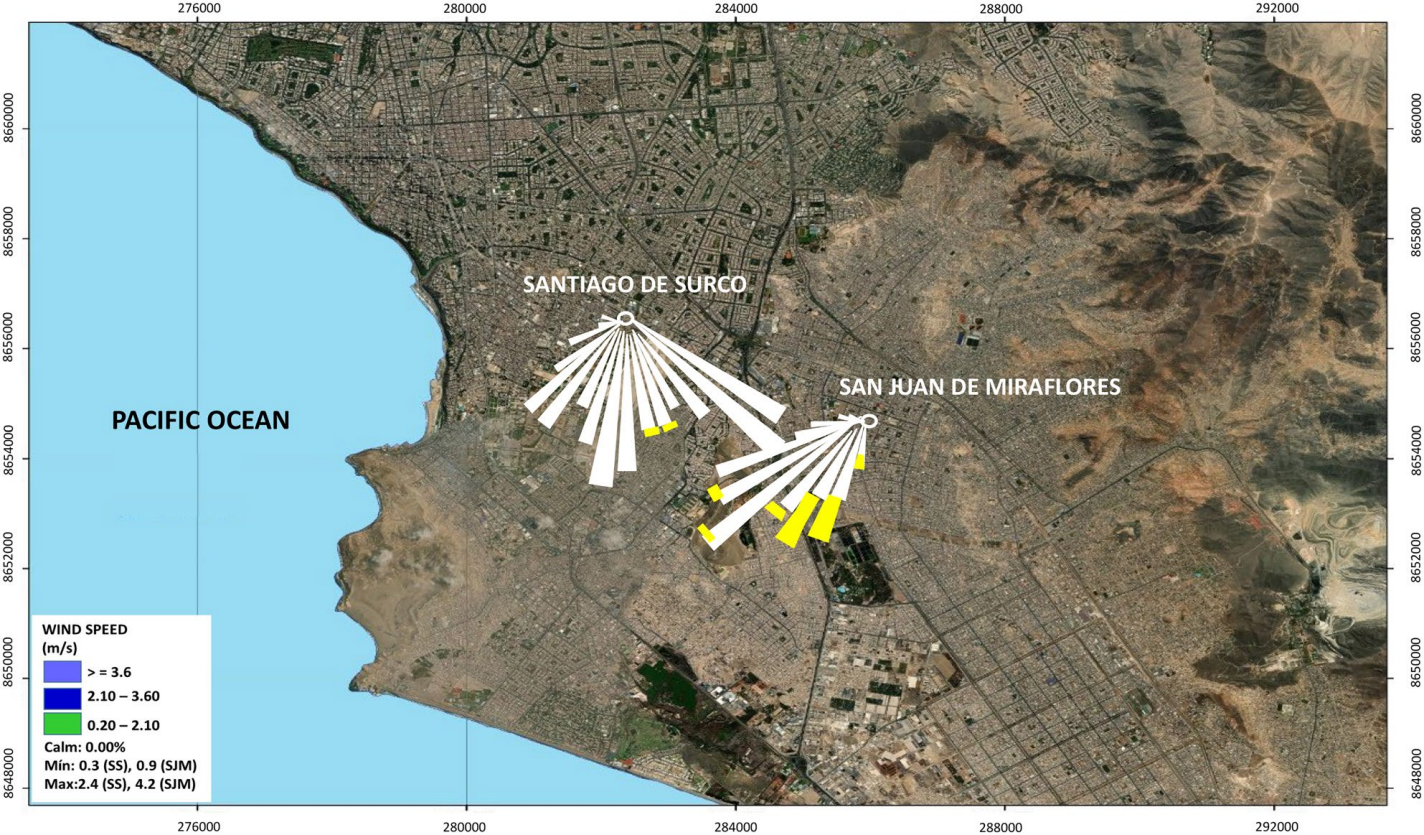
Location map and wind rose for SS and SJM, period 2019–2020. Map created in Arcgis software version 10.4.1 (https://desktop.arcgis.com/es/arcmap/10.4/get-started/setup/arcgis-desktop-quick-start-guide.htm).

The first monitoring station is located at a MINSA facility in the SS district (12$^{\circ }$8’43.47”S 76$^{\circ }$59’50.07”W), and the second is located at the *Hospital María Auxiliadora* in the SJM district (12$^{\circ }$9’41.33”S 76$^{\circ }$57’32.36”W). The monitoring generates continuous information and the publication of real-time data for PM$_{10}$ ($\upmu$g/m$^{3}$), temperature-T ($^{\circ }$C), dew point-DP ($^{\circ }$C), atmospheric pressure-AP (hPa), solar radiation-SR (W/m$^2$), wind speed-WS (m/s) and wind direction-WD (degrees, $^{\circ }$). The map of Lima south-Peru (see Fig. 1) shows the monitoring stations evaluated in the period 2019–2020. This map was prepared using the Arcgis 10.4.1 software, with the shapefiles from Peru, including the Pacific Ocean.

From Fig. 1, both monitoring stations correspond to coastal and surrounding urban areas in southern Lima. The district of *San Juan de Miraflores* is more desert and sandy, with prevailing coastal winds, intense vehicular traffic, dust resuspension^42^ and close to limestone, clay and gypsum quarries. The predominant wind rose presented South (S), South South West (SSW) and South East (SE) directions, with intensities between 0.9 and 2.5 m/s, with a punctual anomalous value in the spring (4.2 m/s) for SJM and between 0.3 to 2.4 m/s for SS.

### Equipment and materials

The calibrated automatic equipment (zero air equipment and argon gas dilution equipment) were installed between 1.5 and 10 m from the ground and connected to 220 volts, avoiding solid barriers 10 meters around to ensure adequate air flow. The Campbell Scientific weather station was calibrated to measure each meteorological variable. Data obtained from January 1, 2019 to December 31, 2020 were processed by real-time telemetry using AirMetReport software. Glass fiber of 25 mm diameter was used, placed daily inside the low volume test equipment (PM$_{10}$ beta gauge) with automatic analysis of PM$_{10}$ concentration in $\upmu$g/m$^{3}$. Monitoring was performed according to the Protocol for Air Quality Monitoring and Data Management^43^. The data were validated by DIGESA and are published on the institutional web page.

### Statistical procedure

A total of 188,859 valid hourly data (T, DP, AP, SR, RH, WD, WS and PM$_{10}$) generated between 2019 and 2020 were used. For 2019 only 83.5% of the data were used, while for 2020 only 51.13% were used. The 2019 data were used to develop the models and their calibration, and the 2020 data were used to validate or evaluate the models obtained in 2019. The hourly scale data were used for plotting temporal variability and statistical analysis. For its part, the wind rose analysis was elaborated using Wrplot View V.8.0.2 software. The input data were used in Origin-Pro 8.0 software, generating the following:

- Analysis of the correlation between meteorological factors and PM$_{10}$.
- Application of PCA and dimensionality reduction of the interrelated variables by linear transformation of the input vectors from high to low dimension, and generation of uncorrelated components by reducing the number of predictor variables through the correlation matrix (M):
  1 $$\begin{aligned} |M-\lambda I| \end{aligned}$$
  where $\lambda$ is an eigenvalue and *I* is the identity matrix, $\lambda$ multiplied by a nonzero eigenvector *E* generates the correspondence ${C_{e}}$
  2 $$\begin{aligned} C_{e}=\lambda E \end{aligned}$$
  Thus, the *j*-th variance of the *j*-th principal component $\text{PC}$ is given as:
  3 $$\begin{aligned} \text{Variance }=\frac{\kappa _{j}}{\Sigma _{n} \lambda _{n}} \end{aligned}$$
  The calculated principal components (PC) generate a maximum $\lambda$ of linear combination of variables with the highest data variability, transforming the original set to the orthogonal one by multiplying the eigenvectors^44^.
- PM$_{10}$ modeling using a single meteorological variable.
- PM$_{10}$ modeling using MLR, defined by:
  4 $$\begin{aligned} Y_{i}=\beta _{0}+\beta _{1} X_{1 i}+\cdots +\beta _{k} X_{k i}+\varepsilon _{i},\quad i=1,2, \ldots , n \end{aligned}$$
  where $Y_i$ the dependent variable; $\beta _0, \beta _1, \ldots , \beta _k$ are $k+1$ constant parameters; $X_1, \ldots , X_k$ are *k* independent variables; $\varepsilon$ are the independent errors identically and Gaussian distributed and *n* is the number of observations^39^.
- Modeling of PM$_{10}$ using the combination of 2 meteorological variables by 3D surface fitting, which is an extension of the ordinary nonlinear fitting for both XYZ and matrix data. The method consisted of converting all data to Log10, assigning the meteorological variables (independent) to the X, Y axes and the PM$_{10}$ (dependent) to the Z axis. The worksheet was converted to matrix using the grid method and random parameters of 24 columns by 24 rows. Different models based on the Levenberg-Marquardt algorithm (damped least squares) were tested taking into account the metrics defined in this item, generating the following functions: Extreme Cum: Non-linear Extreme Value Cumulative Function
  5 $$\begin{aligned} z = z_0 + B * \exp \left\{ -\exp \left\{ \frac{C-x}{D}\right\} \right\} +E * \exp \left\{ -\exp \left\{ \frac{F-y}{G}\right\} \right\} +H \exp \left\{ -\exp \left\{ \frac{C-x}{D}\right\} -\exp \left\{ \frac{F-y}{G}\right\} \right\} \end{aligned}$$
  Voigt2DMod: The voigt surface with volume as parameter, donde: $z_0$, *A*, $x_c$, $w_1$, $y_c$, $w_2$, $m_u$ are constant parameters.
  6 $$\begin{aligned} z=z_{0}+A\left[ m_{u} \frac{4}{\pi ^{2}} * \frac{w_{1}}{4\left( x-x_{c}\right) ^{2}+w_{1}^{2}} * \frac{w_{2}}{4\left( y-y_{c}\right) ^{2}+w_{2}^{2}}+\left( 1-m_{u}\right) \frac{4 L n 2}{\pi w_{1} w_{2}} e^{\frac{-4 L n 2}{w_{1}^{2}}\left( x-x_{c}\right) ^{2}-\frac{-4 L n 2}{w_{2}^{2}}\left( y-y_{c}\right) ^{2}}\right] \end{aligned}$$
  Poly2D: Two-dimensional polynomial function
  7 $$\begin{aligned} z=z_0+a x+b y+c x^{2}+d y^{2}+f x y \end{aligned}$$
  For these tests, 95% reliability, correlation coefficient (r) and coefficient of determination (R$^2$) were considered^45,46^.

### Performance metrics

Analysis of prediction performance involves calculating the errors between the observed and predicted values. Four statistical metrics were used to compare the performance of the models:

1. Pearson correlation coefficient (r):
  8 $$\begin{aligned} \text{r}=\frac{\sum _{i=1}^{n}\left( Y_{o}^{i}-\bar{Y}_{o}\right) \left( Y_{m}^{i}-\bar{Y}_{m}\right) }{\sqrt{\sum _{i=1}^{n}\left( Y_{o}^{i}-\bar{Y}_{o}\right) ^{2}} \cdot \sqrt{\sum _{i=1}^{n}\left( Y_{m}^{i}-\bar{Y}_{m}\right) ^{2}}} \end{aligned}$$
2. Coefficient of determination (R$^{2}$):
  9 $$\begin{aligned} \text{R}^{2}=\frac{\left[ \sum _{i=1}^{n}\left( Y_{o}^{i}-\bar{Y}_{o}\right) \left( Y_{m}^{i}-\bar{Y}_{m}\right) \right] ^{2}}{\sum _{i=1}^{n}\left( Y_{o}^{i}-\bar{Y}_{o}\right) ^{2} * \sum _{i=1}^{n}\left( Y_{m}^{i}-\bar{Y}_{m}\right) ^{2}} \end{aligned}$$
3. Root Mean Squared Error (RMSE):
  10 $$\begin{aligned} \text{RMSE}=\sqrt{\frac{\sum _{i=1}^{n}\left( Y_{m}^{i}-Y_{o}^{i}\right) ^{2}}{n}} \end{aligned}$$
4. Nash-Sutcliffe Efficiency (NSE):
  11 $$\begin{aligned} \text{NSE}=1-\frac{\sum _{i=1}^{n}\left( Y_{m}^{i}-Y_{o}^{i}\right) ^{2}}{\sum _{i=1}^{n}\left( Y_{o}^{i}-\bar{Y}_{o}\right) ^{2}}-\alpha <D C \le 1.0 \end{aligned}$$

where $Y_{o}^{i}, Y_{m}^{i}$, stand for model predicted and target values, respectively, $\bar{Y}_{o}^{i}, \bar{Y}_{m}^{i}$, are their mean values and *n* represents the number of observations.

### Air quality index (AQI) and air quality standards (AQS)

In Peru, the AQI PM$_{10}$ for 24 h (100 $\upmu$g/m$^{3}$) should not be exceeded more than 7 times a year and the annual arithmetic mean should not exceed 50 $\upmu$g/m$^{3}$. The AQI PM$_{10}$ was calculated from the 24-h AQS PM$_{10}$ and the alert threshold value (150 $\upmu$g/m$^{3}$) according to the following expression:

12 $$\begin{aligned} I(\text{PM}_{10})=\frac{\left[ \text{PM}_{10} \upmu \text{g} / \text{m}^{3}\right] 100 \upmu \text{g} / \text{m}^{3}}{150 \upmu \text{g} / \text{m}^{3}} \end{aligned}$$

where *I*(PM$_{10}$) expresses the calculated AQI PM$_{10}$, and the value inside the parenthesis is the observed PM$_{10}$. Table 1 shows the national AQI classification (INCA).

**Table 1:** Air quality index values.

Rating	Care	Values range	Interval ($\upmu$g/m$^{3}$)	Colour
Good	Satisfactory air quality	0–50	0–75	Green
Moderate	Acceptable air quality	51–100	76–150	Yellow
Poor	Health problems are experienced	101-VUEC	151–250	Orange
TVSC*	Causes health effects	> VUEC	> 250	Red

*Threshold value of state of care.

## Results and discussion

### Meteorological variations and seasonal correlations

**Figure 2:**
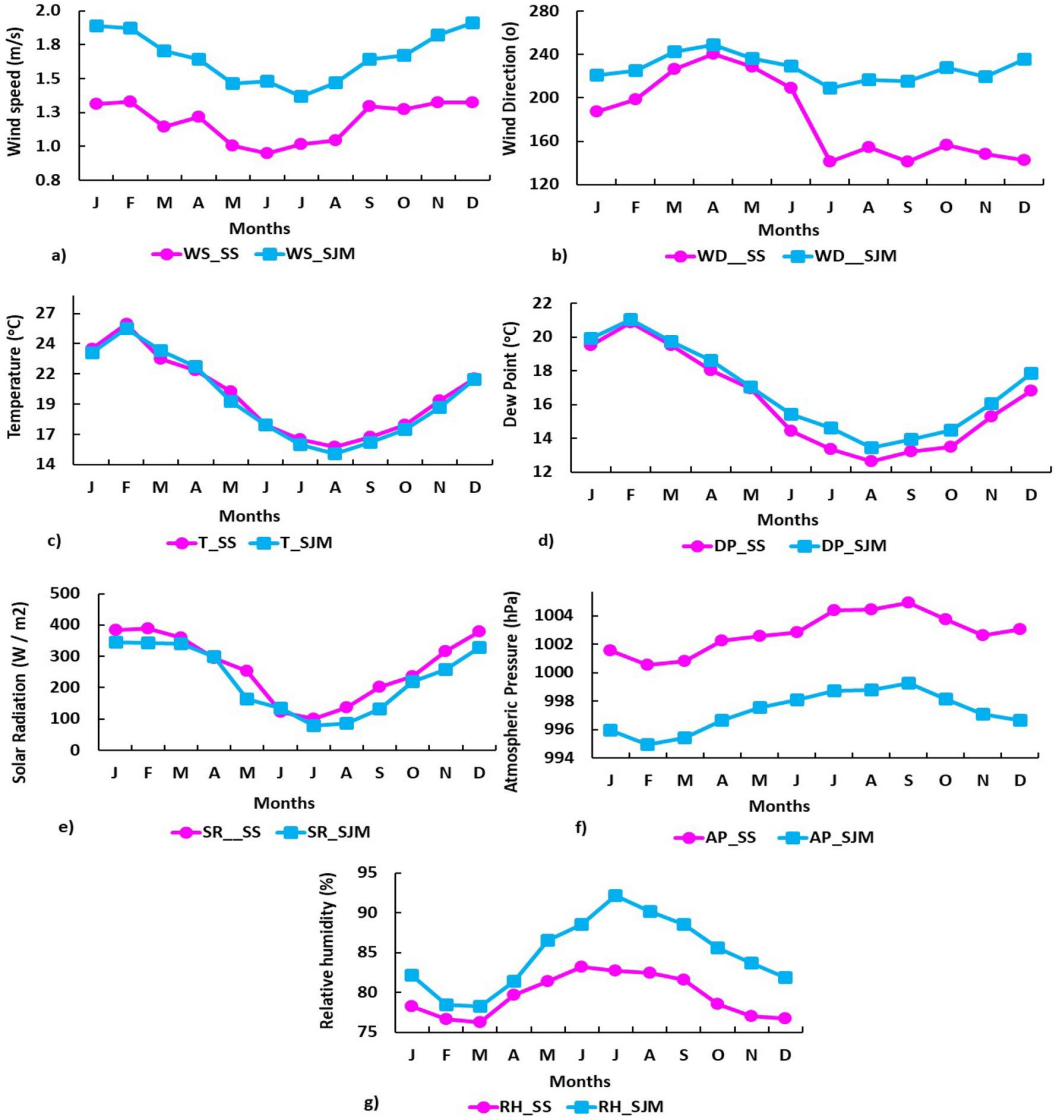
Distribution of the monthly mean values of the meteorological variables at stations SS and SJM, between January and December 2019, Lima-Peru: (**a**) WS (m/s), (**b**) WD (degrees), (**c**) T ($^{\circ }\text{C}$), (**d**) DP ($^{\circ }\text{C}$), (**e**) SR (W/m$^{2}$), (**f**) AP (hPa) and (**g**) RH (%).

Figure 2 shows the average monthly distribution of meteorological variables in SS and SJM during 2019. The monthly averages of meteorological variables evidenced that:

- T, DP and SR variables are higher in the austral summer (January, February and March), while RH and AP have low monthly averages due to the proximity of the sun to the earth.
- In winter, especially in July and August, there is a decrease in SR and T (including DP), and the values of AP and RH increase.

WS varied discretely from $1.06 \,\text{m} / \text{s}$ in January $(\text{SS})$ a $1.93 \,\text{m} / \text{s}$ in December $(\text{SJM})$ (Fig. 2a), with wind patterns in a south-easterly direction for SS and south-westerly for SJM, especially in April (Fig. 2b). Temperature ranged between $15.11^{\circ }\text{C}$ in winter (August-SJM) and $25.56^{\circ }\text{C}$ in summer (February-SS) (ver Fig. 2c), and DP fluctuated between $12.61{ }^{\circ } \text{C}$ (August-SS) and $21.18{ }^{\circ } \text{C}$ (February-SJM) (see Fig. 2d).

**Figure 3:**
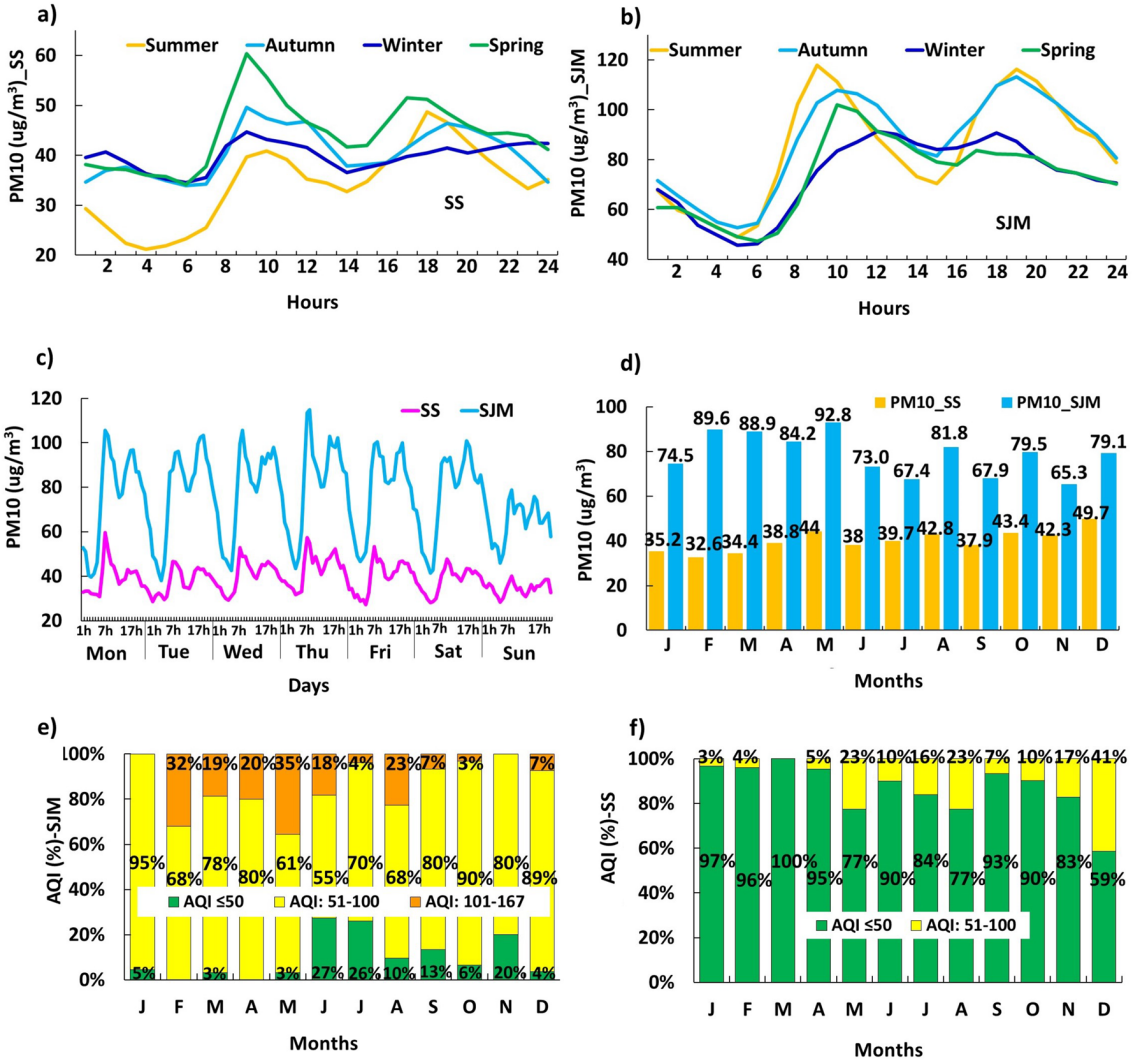
Temporal variation of PM$_{10}$: (**a**) Hourly mean in SS; (**b**) Hourly mean in SJM; (**c**) Daily behavior of PM$_{10}$ throughout the week; (**d**) Monthly mean; (**e**) Percentage of AQI values in SJM; (**f**) Percentage of AQI values in SS. Period 2019.

The annual means of T and DP in both districts showed that $\text{T}_{\text{SS}}>\text{DP}_{\text{SS}}$ (3.65$^{\circ }\text{C}$) and $\text{T}_{\text{SJM}}>\text{DP}_{\text{SJM}}$ (2.59$^{\circ }\text{C}$), but SS was warmer than SJM: $\text{T}_{\text{SS}}>\text{T}_{\text{SJM}}$ (0.26$^{\circ }\text{C}$) and $\text{DP}_{\text{SJM}}>\text{DP}_{\text{SS}}$ (0.68$^{\circ }\text{C}$). Likewise, SR was higher in summer (394.36 $\,\text{W}/\text{m}^{2}$, February-SS) and lower in winter (80.26 $\,\text{W}/\text{m}^{2}$, July-SJM) (Fig. 2e), with annual means $\text{SR}_{\text{SS}}>\text{SR}_{\text{SJM}}$ (34.1 $\,\text{W}/\text{m}^{2}$) indicating that the Peruvian central coastal strip presents greater annual variations of solar energy received over the surface^47^. In contrast, AP (see Fig. 2f) and RH (see Fig. 2g) showed an inverse behavior and particular patterns ($\text{RH}_{\text{SS}}<\text{RH}_{\text{SJM}}$ and $\text{AP}_{\text{SS}}>\text{AP}_{\text{SJM}}$), with mean monthly values of RH ranging between 69.8% (Summer-March, SS) and 91.94% (Winter-July, SJM), while AP fluctuated between 994.99 hPa (February, SJM) and 1004.73 hPa (September, SS), with maximum peaks in the winter months. Figure 3 shows the variability of meteorological factors in southern Lima, representing the temporal evolution of PM$_{10}$ in *Santiago de Surco* and *San Juan de Miraflores* during 2019.

### Hourly, daily, weekly and monthly variation of PM$_{10}$ concentration

Figure 3a,b, show lower PM$_{10}$ hourly averages in the early morning (1:00 a.m. and 5:00 a.m.), associated with higher humidities and weaker winds that kept aerosols suspended. As the hours passed, $\text{T}$ increased and RH decreased, concentrating PM$_{10}$ in the air column, especially at peak hours in the morning (9:00 a.m. and 12:00 p.m.) and at night (6:00 p.m. and 9:00 p.m.). In SS, PM$_{10}$ at peak hours followed the seasonal order: spring $\left( 60.38 \upmu \text{g} / \text{m}^{3}\right)>$ autumn $\left( 49.64 \upmu \text{g} / \text{m}^{3}\right)>$ winter $\left( 44.73 \upmu \text{g} / \text{m}^{3}\right) ,>$ summer $\left( 40.92 \upmu \text{g} / \text{m}^{3}\right) \text{y}$ and in $\text{SJM}$: summer $\left( 117.9 \upmu \text{g} / \text{m}^{3}\right)>$ autumn (107.8 $\left. \upmu \text{g} / \text{m}^{3}\right)>$ spring $\left( 102.1 \upmu \text{g} / \text{m}^{3}\right)>$ winter $\left( 91.3 \upmu \text{g} / \text{m}^{3}\right)$. The intense activity of the Lima-Callao vehicle fleet^48^, dust resuspension and industrial activities generate this effect.

Likewise, Fig. 3c shows the daily averages of PM$_{10}$, in SS and SJM. Lower values were recorded on Saturdays and Sundays (SS: $29 \upmu \text{g} / \text{m}^{3}$ and $\text{SJM}: 54.58 \upmu \text{g} / \text{m}^{3}$ ), due to formal and student work breaks, and decreased activity of vehicular mobile sources^48^. For the other days of the week, the values were higher (Wednesday in SS: 46 $\upmu \text{g} / \text{m}^{3}$, in spring; and Monday in SJM: 97.94 $\upmu \text{g} / \text{m}^{3}$, in autumn).

On the other hand, the monthly mean values for PM$_{10}$ (Fig. 3d) ranged from 32.6 $\upmu \text{g} / \text{m}^{3}$ (February-SS) to 92.8 $\upmu \text{g} / \text{m}^{3}$ (May-SJM). Post hoc tests produced for SJM a significant difference $(\text{p}=0.0)$ between concentrations recorded during the warm austral summer-autumn months (PM$_{10}$: 89.6 to 92.8 $\upmu \text{g} / \text{m}^{3}$ ) and cool winter-spring months (PM$_{10}$: 73 to 79.1 $\upmu \text{g} / \text{m}^{3}$. The decrease in TI base altitude in the coastal summer-autumn coincided with the highest PM$_{10}$ concentrations.

### Air quality indexes

In 2019, the AQIs values in South Lima (Fig. 3e,f) were 49.3 $\%$ good quality $\left( \text{PM}_{10}<76 \upmu \text{g} / \text{m}^{3}\right) >44.1 \%$ moderate quality $\left( 76-150 \upmu \text{g} / \text{m}^{3}\right) >6.5 \%$ poor quality (101-250 $\upmu \text{g} / \text{m}^{3}$ threshold state of care). In addition, differences were found between SS and SJM AQIs. The AQI$_{\text{SJM}}$ values were $77 \%$ of moderate quality $>14 \%$ of poor quality $>8.8 \%$ of good quality, while the AQI$_{SS}$ values were $86 \%$ of good quality $>14 \%$ of moderate quality. On the other hand, $13 \%$ of hourly $\text{PM}_{10\text{SS}}$ values exceeded the WHO reference value $\left( 50 \upmu \text{g} / \text{m}^{3}\right)$ but their annual mean $40 \upmu \text{g} / \text{m}^{3}$ did not exceed the WHO annual reference value ($50 \upmu \text{g} / \text{m}^{3}$ ); while for $\text{SJM}$, $100 \%$ of hourly data exceeded the WHO reference. Likewise, $15 \%$ of hourly $\text{PM}_{10\text{SJM}}$ values exceeded the current national AQS $\left( 100 \upmu \text{g} / \text{m}^{3}\right)$ and the annual mean $\left( 78.7 \upmu \text{g} / \text{m}^{3}\right)$ exceeded the annual AQS $\left( 50 \upmu \text{g} / \text{m}^{3}\right)$. The fact is that both locations present a potential risk to respiratory and cardiovascular diseases, especially SJM due to the daily exposure of people to higher PM$_{10}$ levels.

### Meteorological and PM$_{10}$ variations in 2020 during the COVID-19 pandemic

**Figure 4:**
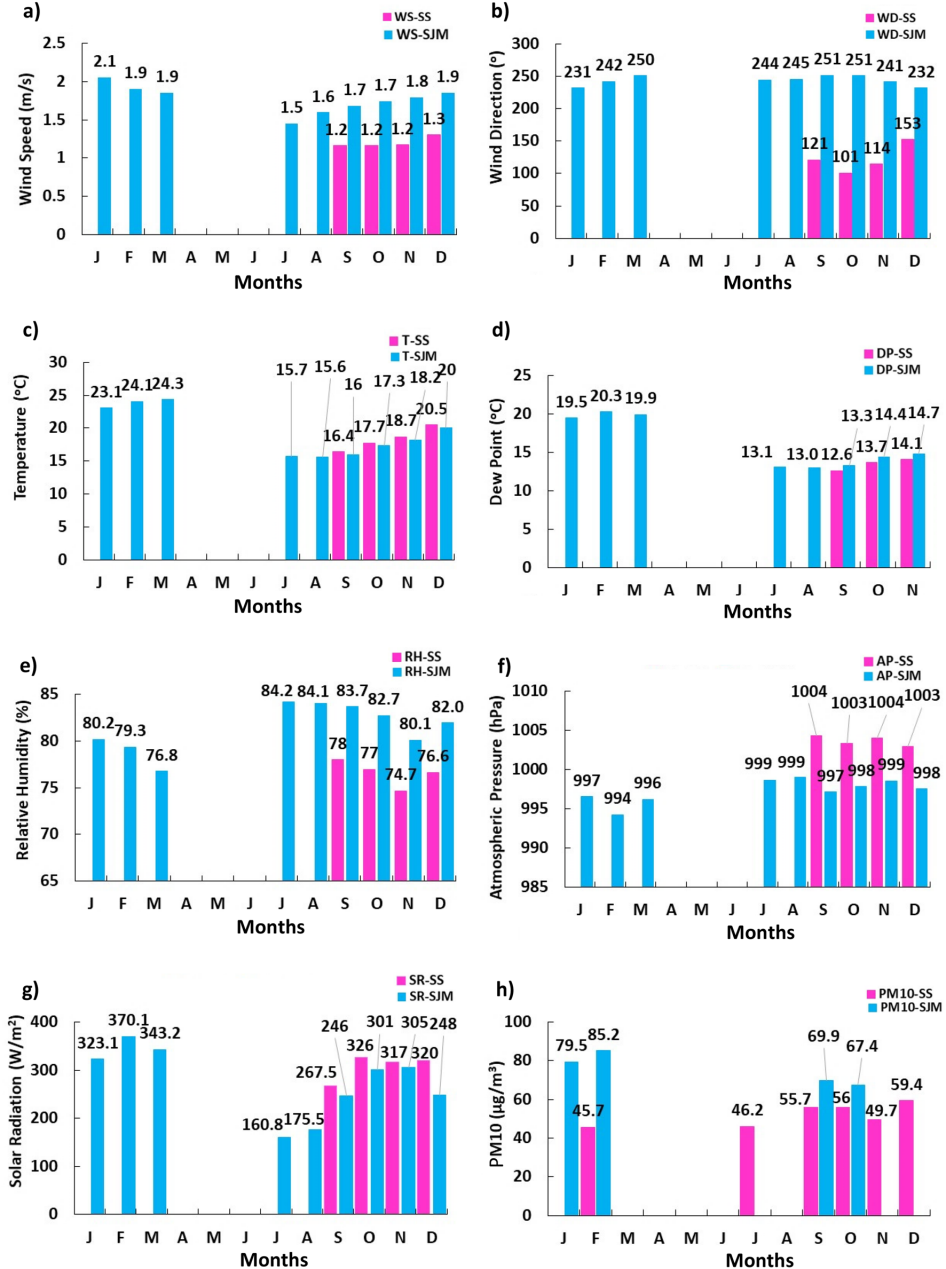
Distribution of the monthly mean values of the meteorological variables at the SS and SJM stations, between January and December 2020: (**a**) WS (m/s), (**b**) WD (degrees), (**c**) T ($^{\circ }\text{C}$), (**d**) DP ($^{\circ }\text{C}$), (**e**) AP (hPa) and (**f**) RH (%), (**g**) SR (W/m$^2$) and (**h**) PM$_{10}$ ($\upmu$g/m$^{3}$).

Due to the pandemic, annual monitoring of meteorological variables in SJM was suspended between April and June and PM$_{10}$ was monitored in January, February, September and October. In SS, meteorological monitoring was continuous and PM$_{10}$ was monitored in February and July-December. On the other hand, the increase in monthly average RH in South Lima was notorious, as $\text{SR}_{\text{SJM2020}}(274.74 \,\text{W}/\text{m}^{2})>\text{SR}_{\text{SJM2019}}(227.37 \,\text{W}/\text{m}^{2})$ and $\text{SR}_{\text{SS2020}}$ $\left( 307.68 \,\text{W} / \text{m}^{2}\right) >\text{SR}_{\text{SS} 2019}\left( 258.68 \,\text{W} / \text{m}^{2}\right)$. The wind pattern for SS had dominant east-southeast direction and for SJM southwest direction. In 2020, the comparison of PM$_{10}$ concentrations for SJM corresponding to the months of each year showed no significant differences, while for SS a 25% increase PM$_{10}$ concentration was observed. The pandemic did not produce reduced PM$_{10}$ levels in 2020 as expected due to the operability of major emission sources during that time^49^. The monitoring results are shown in Fig. 4.

### Correlations between meteorological variables and PM$_{10}$

**Figure 5:**
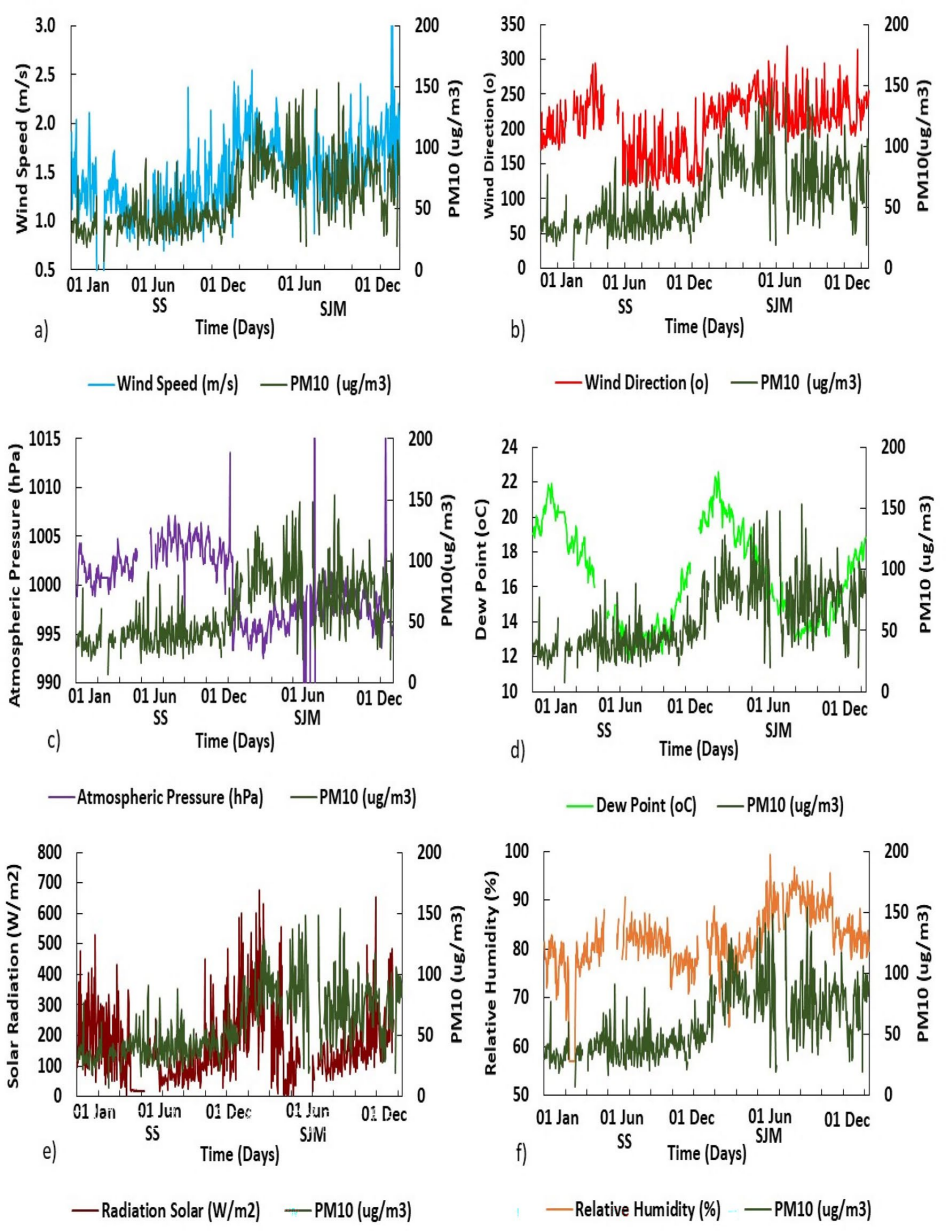
Daily variations of meteorological variables and concentrations of PM$_{10}$, for South Lima in 2019. (**a**) WS, (**b**) WD, (**c**) AP, (**d**) DP, (**e**) SR and (**f**) RH.

**Figure 6:**
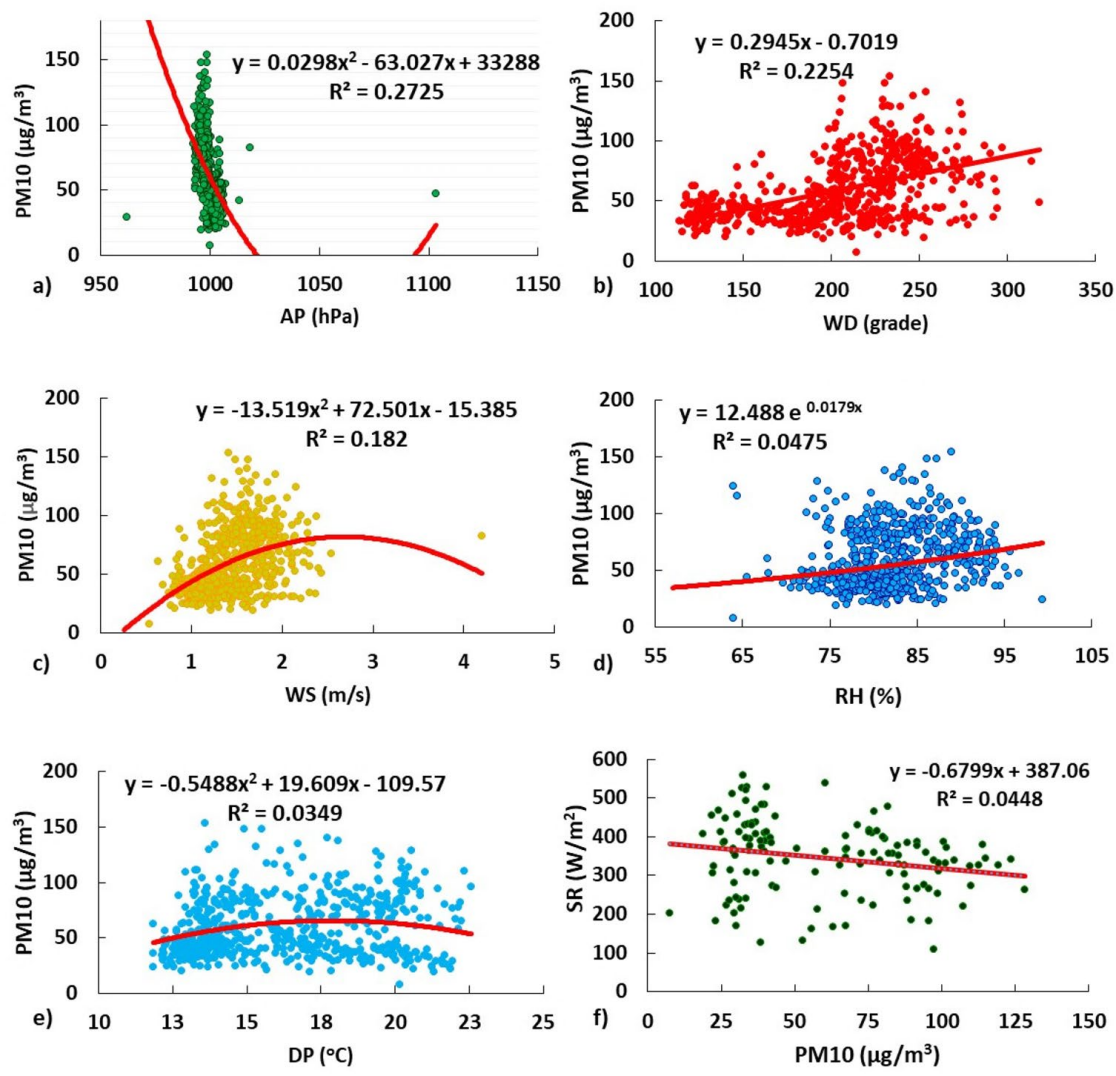
Regressions of a single meteorological factor $(p < 0.05)$ and PM$_{10}$ in South Lima in 2019: (**a**) AP-PM$_{10}$ Annual, (**b**) WD-PM$_{10}$ Annual, (**c**) WS-PM$_{10}$ Annual, (**d**) RH-PM$_{10}$ Annual, (**e**) DP-PM$_{10}$ Annual, (**f**) PM$_{10}$-SR (summer).

Table 2, shows the correlations determined for the meteorological variables and PM$_{10}$ data observed in 2019. Significant statistical values $(\text{p}<0.05)$, with strong and moderate magnitudes, are shown in bold: r$_\text{T-DP}$ $(0.95788)>\text{r}_{\text{T-SR}}$ $(0.72471)>\text{r}_{\text{RH-SR}}(-0.66936)>\text{r}_{\text{RH-T}}(-0.6484)>\text{r}_{\text{DP-SR}}(0.61907)$. Regarding PM$_{10}$, the order was moderate and weak: ${\text{r}_\text{WD}}-{\text{PM}_{10}}$ $(0.48192)>\text{r}_{\text{WS}-\text{PM}_{10}}(0.40526)>\text{r}_{\text{AP}-\text{PM}_{10}}(-0.39443)$ $>\text{r}_{\text{RH}-\text{PM}_{10}}(0.18348)>\text{r}_{\text{DP}-\text{PM}_{10}}(0.13796)$. Likewise, Fig. 5 shows the fluctuations of each meteorological variable and PM$_{10}$ concentrations. While, Fig. 6 presents the single variable regressions in Cartesian coordinates for southern Lima in the period 2019.

**Table 2:** Pearson correlations of the meteorological variables and the annual and seasonal PM$_{10}$.

	RH	T	DP	AP	SR	WS	WD	PM_10_
**Summer**								
RH	1	− 0.67751	0.056	− 0.33923	− 0.26431	0.66721	0.2313	0.02371
T	− 0.67751	1	0.64416	− 0.05572	0.34623	0.04159	− 0.15641	0.08682
DP	0.056	0.64416	1	− 0.27044	0.15988	0.08832	0.02631	0.07581
AP	− 0.33923	− 0.05572	− 0.27044	1	0.13959	− 0.59284	− 0.59514	− 0.74682
SR	− 0.26431	0.34623	0.15988	0.13959	1	0.09111	− 0.11085	− 0.21155
WS	0.66721	0.04159	0.08832	− 0.59284	0.09111	1	0.01221	0.55296
WD	0.2313	− 0.15641	0.02631	− 0.59514	− 0.11085	0.01221	1	0.55989
PM$_{10}$	0.02371	0.08682	0.07581	− 0.74682	− 0.21155	0.55296	0.55989	1
**Autumn**								
RH	1	− 0.80644	− 0.49657	− 0.3199	− 0.67986	− 0.0763	0.21508	0.22361
T	− 0.80644	1	0.90638	0.09043	0.81919	0.30554	− 0.13793	− 0.04332
DP	− 0.49657	0.90638	1	0.05445	0.74073	0.39667	− 0.00969	0.10543
AP	− 0.3199	0.09043	0.05445	1	0.15268	− 0.42613	− 0.07857	− 0.72994
SR	− 0.67986	0.81919	0.74073	0.15268	1	0.43466	− 0.14084	0.01694
WS	− 0.0763	0.30554	0.39667	− 0.42613	0.43466	1	− 0.06862	0.56274
WD	0.21508	− 0.13793	− 0.00969	− 0.07857	− 0.14084	− 0.06862	1	0.08837
PM$_{10}$	0.22361	− 0.04332	0.10543	− 0.72994	0.01694	0.56274	0.08837	1
**Winter**								
RH	1	− 0.41628	0.58515	− 0.16319	− 0.55647	0.39848	0.54395	0.35943
T	− 0.41628	1	0.49301	0.12102	0.38446	0.04495	− 0.18351	− 0.06938
DP	0.58515	0.49301	1	− 0.0305	− 0.20124	0.42113	0.35397	0.2957
AP	− 0.16319	0.12102	− 0.0305	1	0.05873	− 0.07583	− 0.24061	− 0.23791
SR	− 0.55647	0.38446	− 0.20124	0.05873	1	0.02819	− 0.05578	0.14091
WS	0.39848	0.04495	0.42113	− 0.07583	0.02819	1	0.23279	0.29453
WD	0.54395	− 0.18351	0.35397	− 0.24061	− 0.05578	0.23279	1	0.5149
PM$_{10}$	0.35943	− 0.06938	0.2957	− 0.23791	0.14091	0.29453	0.5149	1
**Spring**								
RH	1	− 0.48193	0.04568	− 0.47784	− 0.53996	0.26821	0.54183	0.39192
T	− 0.48193	1	0.85274	− 0.03936	0.43651	0.21912	− 0.0587	− 0.04819
DP	0.04568	0.85274	1	− 0.33073	0.1734	0.41321	0.2611	0.184
AP	− 0.47784	− 0.03936	− 0.33073	1	0.05839	− 0.25586	− 0.71338	− 0.54587
SR	− 0.53996	0.43651	0.1734	0.05839	1	0.23968	0.00886	0.14659
WS	0.26821	0.21912	0.41321	− 0.25586	0.23968	1	0.40479	0.33795
WD	0.54183	− 0.0587	0.2611	− 0.71338	0.00886	0.40479	1	0.72592
PM$_{10}$	0.39192	− 0.04819	0.184	− 0.54587	0.14659	0.33795	0.72592	1
**Annual**								
RH	1	− 0.64912	− 0.44149	− 0.08189	− 0.67403	0.23093	0.23457	0.18833
T	− 0.64912	1	0.95778	− 0.21475	0.7253	0.28424	0.22679	0.05294
DP	− 0.44149	0.95778	1	− 0.257	0.6178	0.20475	0.36127	0.12721
AP	− 0.08189	− 0.21475	− 0.257	1	− 0.08977	− 0.24426	− 0.37568	− 0.38676
SR	− 0.67403	0.7253	0.6178	− 0.08977	1	0.34519	0.10545	0.04713
WS	0.23093	0.28424	0.20475	− 0.24426	0.34519	1	0.17999	0.39432
WD	0.23457	0.22679	0.36127	− 0.37568	0.10545	0.17999	1	0.47482
PM$_{10}$	0.18833	0.05294	0.12721	− 0.38676	0.04713	0.39432	0.47482	1

The wind speed and direction presented in Fig. 5a, shows the average daily fluctuation of WS in southern Lima, with a U-shaped distribution for SS and SJM, and Fig. 5b shows the fluctuation for WD. Seasonally, the range of velocities was lower for SS $\text{SS}(1.15-1.34 \,\text{m} / \text{s})$ and higher for SJM (1.49-1.86 m/s). Regarding the WS-PM$_{10}$ relationship, some authors calculated negative correlations^13,14^. On the contrary, positive and direct correlations were calculated (r$_{\text{WS-PM}_{10}}-\text{anual}(0.40526)$), especially for summer (r$_{\text{WS-PM}_{10}}(0.55296)$) and autumn (r$_{\text{WS-PM}_{10}}(0.56274)$). These values are higher than other studies reported in Lima^34^, indicating influence on PM$_{10}$ dispersion, resuspension and transport, including its decrease with the simultaneous diminution of wind^50^. The non-parametric regression produced a coefficient of determination $\left( \text{R}^{2}=0.182\right)$, close to other studies on the same variable^13^ (Fig. 6c). On the other hand, with respect to annual WD in southern Lima, the correlations were also significant r$_{\text{WD-PM}_{10}}(0.48192)$ (Fig. 5b), in the order: $\text{r}_{\text{spring}}$ $(0.72592)>$ $\text{r}_{\text{ summer }}(0.55989)>\text{r}_{\text{ winter }}(0.5149)>\text{r}_{\text{ autumn }}(0.08837)$.

Regarding the atmospheric pressure presented in Fig. 5c, shows higher AP values for lower PM$_{10}$ values, confirmed by their negative correlation in the order: summer ($\text{r}_{\text{AP}-\text{PM} 10}=-0.74682)>$ autumn $(\text{r}_{\text{AP}-\text{PM} 10}=-0.72994)>$ spring $\left( \text{r}_{\text{AP}-\text{PM} 10}=-0.54587\right)>$ annual $\left( \text{r}_{\text{AP}-\text{PM} 10}=-0.39443\right)$ (see Table 2). In that sense, Li points out that low atmospheric pressures associated with downward mass fluxes restrict the upward movement of PM by accumulating them in the air column^14^. The AP-PM$_{10}$ statistical adjustment produced a weak significant regression $\left( \text{R}^{2}=0.2725, \text{p}<0.05\right)$, being a stronger relationship calculated with respect to the other variables.

On the other hand, temperature, dew point and relative humidity were also evaluated in this study. Contrary to Govindasamy’s study, no significant correlations were generated for T-PM$_{10}$, but significant correlations were generated for DP-PM$_{10}$^49^. These were weak and direct in winter ($\left. \text{r}_{\text{DP}-\text{PM} 10}=0.2957\right)$ and spring ($\left. \text{r}_{\text{DP}-\text{PM} 10}=0.184\right)$, and the regressions were also weak $\left( \text{R}^{2}=0.0349\right)$ (Fig. 6e). According to Szep, if T $>\text{DP}$, as occurs in southern Lima, the pollutant concentration and atmospheric stability decrease causing dilution of the pollutant and its partial wet precipitation^40^. The SS zone presents a greater $\text{T}-\text{DP}\left( \text{SS}:3.65^{\circ } \text{C}\right)$ difference than ${\text{SJM}}\left( \text{SJM}:2.59^{\circ } \text{C}\right)$ which would favor a greater dilution of PM$_{10}$.

On the other hand, it is evident that the high RH in Lima in winter is associated with haze and rainfall events, generating drifting particulate matter (PM$_{10}$), that is removed from the air column by wet precipitation^40,51^. The warm periods (summer) do not usually present rainfall and RH decreases as a characteristic of desert geography, generating a significant T-RH correlation ($\text{r}_\text{T-RH}-\text{summer}=0.67751$ and $\text{r}_\text{T-RH}-\text{summer}=0.80644$). A more stable atmosphere with progressive decrease in the altitude of the TI base in January-May (warm periods) produced greater evaporation and accumulation of PM$_{10}$ especially in SJM. The RH-PM$_{10}$ correlation presented the following order: autumn ($\left. \text{r}_{\text{RH}-\text{PM} 10}=0.22361\right)>$ spring ($\text{r}_{\text{RH}-\text{PM} 10}=0.39192$ ) > winter $\left( \text{r}_{\text{RH}-\text{PM} 10}=0.35943\right)$, which generated a weak regression $\left( \text{R}^{2}=0.0475\right.$, Fig. 6d) as found in other international studies ($\text{r}_{\text{RH}-\text{PM} 10}=0.382$ to 0.467)^15^.

In addition, about the solar radiation presented in Fig. 4g shows a marked variability in the trends between SR and PM$_{10}$, with a weak inverse correlation in summer $(\text{r}=-0.21155)$. This led to calculate particular correlations in each zone according to the seasons of the year, among them: summer $\left( \text{r}_{\text{SS}}=0.15194\right.$; $\left. \text{r}_{\text{SJM}}=0.009844\right)$, autumn $\left( \text{r}_{\text{SS}}=0.03957 ; \quad \text{r}_{\text{SJM}}=0.124166\right) , \quad$ winter $\quad \left( \text{r}_{\text{SS}}=0.28932\right.$, $\left. \text{r}_{\text{SJM}}=0.5552\right)$ and spring $\left( \text{r}_{\text{SS}}=0.33422 ; \quad \text{r}_{\text{SJM}}=0.44675\right)$. The results were congruent with those reported in other investigations; for example, Vardoulakis and Kassomenos, also reported weak correlations in European cities during warm $(\text{r}=$ 0.02 to 0.06) and cool $(\text{r}=$ 0.11 to 0.38) months^52^. On the other hand, direct combinations of high PM$_{10}$-SR or low PM$_{10}$-SR values would be infrequent and suggest that high PM$_{10}$ concentrations could decrease RH intensity^53^. Indeed, the study showed reductions in RH in this coastal area of the southern solstice circle^54^. being between $11.5\%$ (summer) and $25.3 \%$ (winter) for elevated seasonal averages of PM in SJM (73.14 to $87.21 \upmu \text{g} / \text{m}^{3}$ ) compared to SS, and mineral dust aerosols scatter and absorb some of the RH reaching land^53^.

### Multivariate relationships using the PCA

Table 3 shows the principal component analysis. Eigenvalues were produced for three principal components ($\text{PC}$) that explained between 80% and 88% of the total variance of PM$_{10}$ concentrations. As the PC factor is the square of the factor loading, it has been interpreted as the equivalent of the coefficient of determination. The PM$_{10}$ variances in summer $(83\%)$, autumn $(88\%)$, winter $(80\%)$ and spring $(88\%)$ showed moderate loadings of the variables with only one factor^55^.

**Figure 7:**
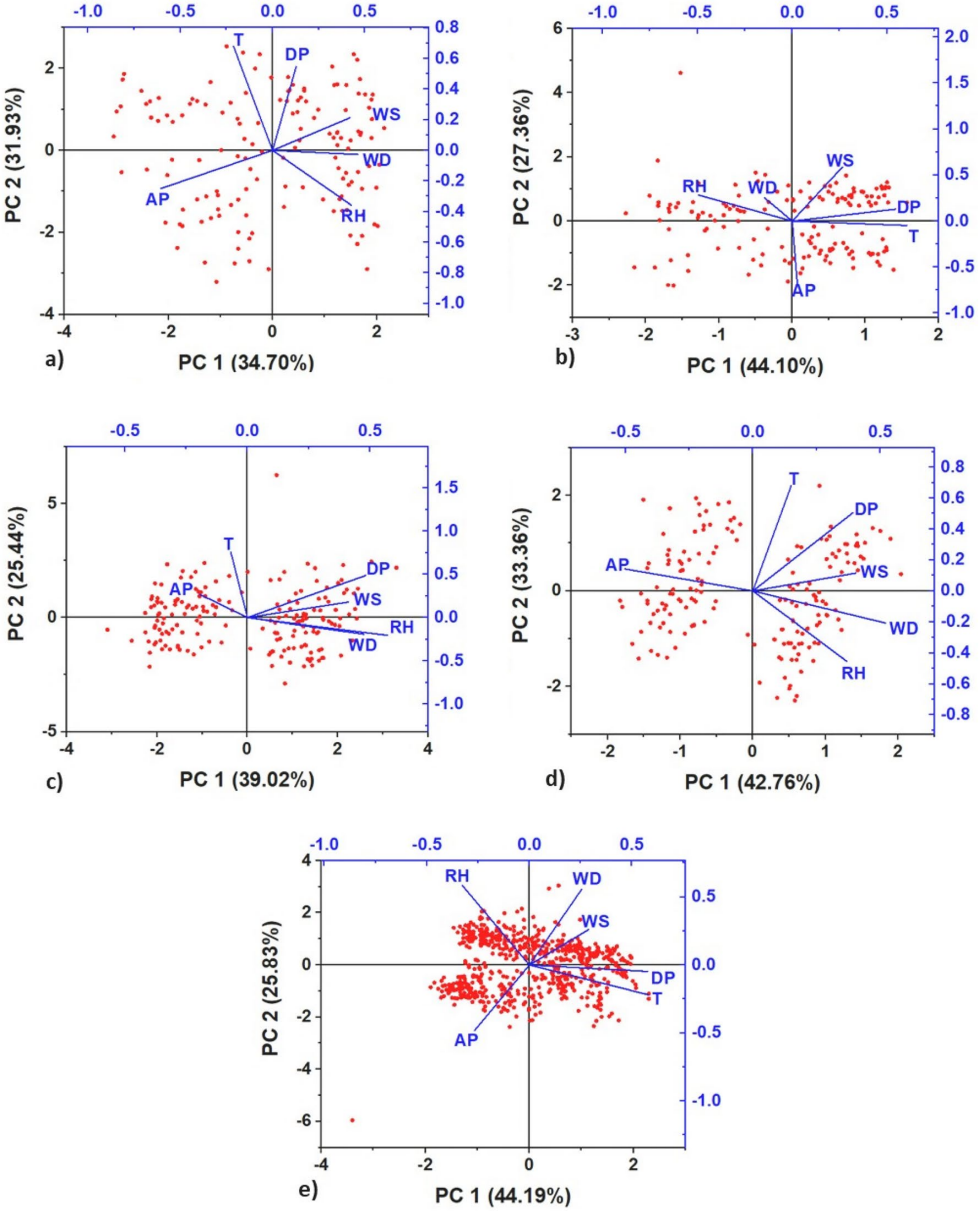
Principal components analysis: (**a**) summer, (**b**) autumn, (**c**) winter, (**d**) spring, (**e**) annual.

In summer, factors PC1 (RH-AP) and PC2 (T-DP) explained $67\%$ of the variance, with descending wind flows and low pressure levels favoring increased humidity and atmospheric stability and decreasing the altitude of the TI base and thermal gradients (1.1 to $0.9^{\circ } \text{C} / 100 \,\text{m}$), increasing PM$_{10}$^56^. In autumn, factors PC1 (RH-T-DP) and PC2 (AP-WS) explained $71\%$ of the variance, witnessing in May atmospheric stability that prevented vertical development of the mixing layer and maintained high PM$_{10}$ levels, but in June coastal winds, humidity, thermal gradient $\left( 2.5^{\circ } \text{C} / 100 \,\text{m}\right)$ and TI base altitude (756.6 m) intensified^56^, driving aerosol dispersion. In winter, factors PC1 (RH-WS-WD) and PC2 (T-DP) explained 64% of the variance, with higher thermal gradients $\left( 2.6^{\circ } \text{C}-3.4^{\circ } \text{C} / 100 \,\text{m}\right)$, wind intensity, humidity and TI base altitude $(>750 \,\text{m})$ favoring higher PM$_{10}$ dispersion^56^. In spring, factors PC1 (AP-WD) and PC2 (RH-T-DP), explained $76\%$ of the variance, producing greater atmospheric instability without significant humidity inputs and temperature increases that warmed the surface and favored the dispersion of the pollutant^56^. Figure 7 shows the 2 main factors (PC1 and PC2) that concentrated the highest percentages of the PCA in the seasons of the year for 2019.

**Table 3:** Principal Components of meteorological variables. EV: Eigenvalue (%); PV: Percentage of variance (%) and CV: Cumulative variance.

	Summer			Autumn			Winter			Spring			Annual		
	PC1	PC2	PC3	PC1	PC2	PC3	PC1	PC2	PC3	PC1	PC2	PC3	PC1	PC2	PC3
EV	2.1	2	1	2.6	1.6	1	2.3	1.5	0.9	2.6	2	0.7	2.7	1.5	0.8
PV	35	32	16	44	27	16	39	25	15	43	33	12	44	26	14
CV	35	67	83	44	71	88	39	64	80	43	76	88	44	70	84
RH	0.43	− 0.4	− 0.2	− 0.5	0.3	− 0.02	0.57	− 0.2	0.25	0.37	− 0.45	0.14	− 0.3	0.58	0
T	− 0.21	0.7	0.1	0.6	− 0.1	0.15	− 0.07	0.76	− 0.31	0.15	0.68	− 0.14	0.58	− 0.22	− 0.1
DP	0.13	0.6	0.1	0.5	0.1	0.2	0.48	0.49	− 0.02	0.39	0.5	− 0.08	0.58	− 0.05	− 0.13
AP	− 0.6	− 0.3	− 0.1	0	− 0.7	0.1	− 0.2	0.27	0.9	− 0.5	0.14	0.5	− 0.3	− 0.48	0.01
WS	0.42	0.2	− 0.7	0.3	0.6	− 0.3	0.41	0.18	0.16	0.4	0.12	0.83	0.29	0.26	0.87
WD	0.46	0	0.7	− 0.2	0.3	0.92	0.48	− 0.19	− 0.1	0.52	− 0.21	− 0.16	0.25	0.55	− 0.45

### Multiple regression model

The meteorological and PM$_{10}$ data observed in 2019 produced the following multiple regression model with significant $\text{p}$-value $(\text{p}<0.05)$:

13 $$\begin{aligned} \text{PM}_{10}=612.9611+2.90988\text{RH}+14.68703\text{T}-16.8064\text{DP}-0.88883\text{AP}+22.90704\text{WS}+0.251\text{WD} \end{aligned}$$

The relative error for the intercept was equal to 217.335 and for the coefficients of the variables ranged from 0.16989 (AP) to 6.5235 (DP). The determination factor reflected a weak fit $\left( \text{R}^{2}=0.3802\right)$, evidencing the limitations of the method. This result was consistent with the model described by Lin^38^, for the T-WS-PM$_{10}$ combination $\left( \text{R}^{2}=0.394\right)$. In contrast, Ceylan did not obtain a significant model in similar tests^39^. Likewise, Kamarul related RH-T with a MODIS-AOD550 satellite factor to improve the regression $\left( \text{R}^{2}=0,66\right)$, but suggested optimizing it and including WS-WD^16^.

### Three-dimensional models

**Figure 8:**
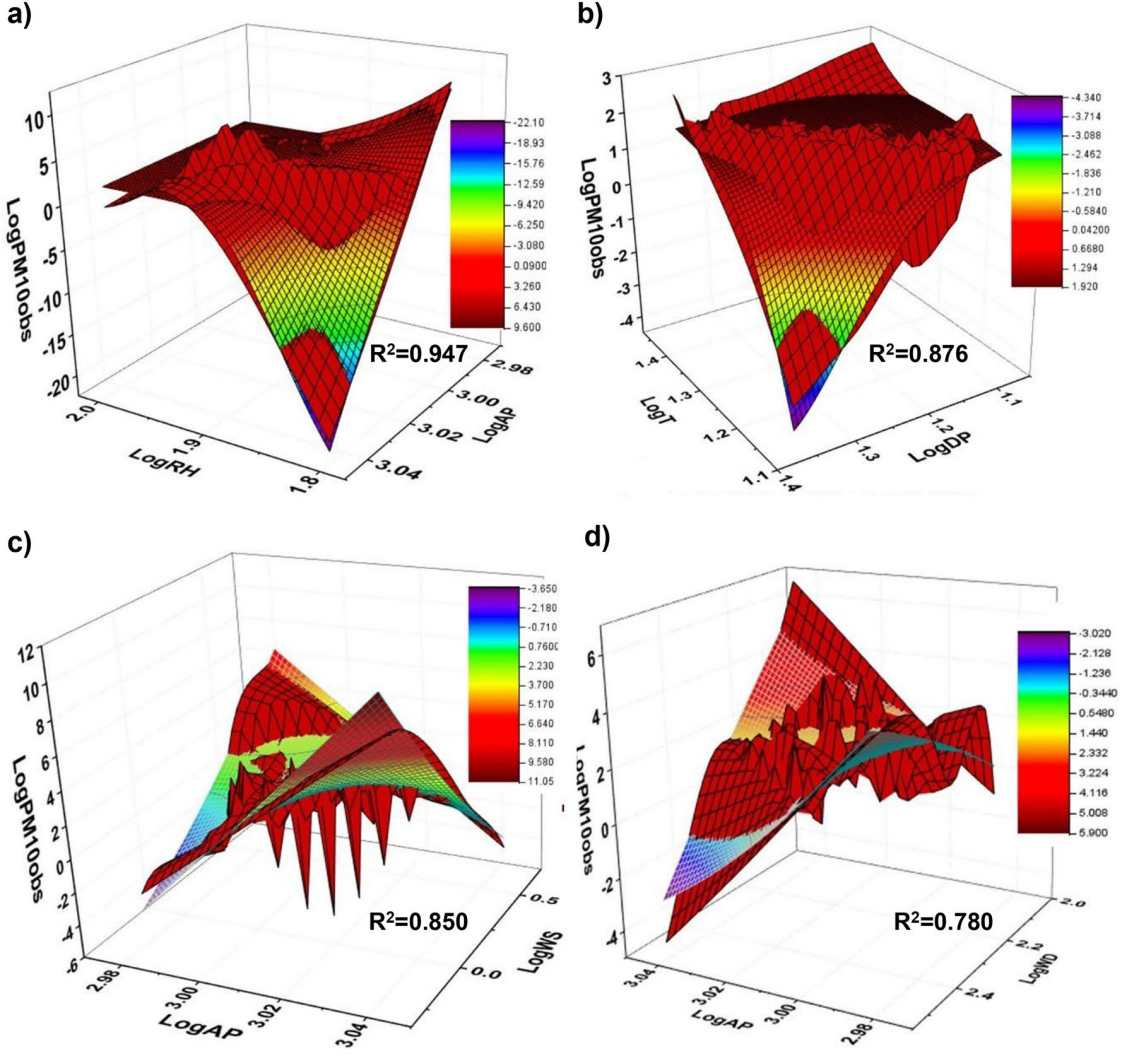
Functional relationships for meteorological bi-variable combinations with PM$_{10}$ concentration, expressed as: (**a**) logRH-logAP, (**b**) logT-logDP, (**c**) logAP-logWS and (**d**) logAP-logWD.

The multiple variables integrally influence the dilution and diffusion of atmospheric pollutant^13^. Under this assumption, statistical fitting of 3D surfaces was performed. The results yielded strong and significant regressions $\left( \text{R}^{2}>0.75; \text{p}<0.05\right)$, in the order: $\text{RH}-\text{AP}-\text{PM}_{ 10}\left( \text{R}^{2}=0.94685\right.$, Fig. 8a) $>\text{T}-\text{DP}\left( \text{R}^{2}=0.87646\right.$, Fig. 8b $)>\left( \text{AP}-\text{WS}-\text{PM}_{10}, \text{R}^{2}=0.85064\right.$, Fig. 8c) $>(\text{AP}-\text{WD}-$ $\text{PM}_{10}, \text{R}^{2}=0.77984$, Fig. 8d). These three-dimensional models (see Table 4) correspond with the results obtained in the PCA and explain that relative humidity and atmospheric pressure largely affect the PM$_{10}$ concentration, as well as temperatures and wind action. Compared with the curve fitted under the influence of a single factor and MLR, the fitting performance of the functional relationship is higher and confirms that different meteorological factors have different effects on PM$_{10}$ concentration.

**Table 4:** Three-dimensional models.

Model	Notation
M1	$z= -0.29-311.14e^{-e^{-(x-1.85)/(-0.05)}}+3.17e^{-e^{-(y-3.15)/(-0.14)}}+428.35e^{-e^{-(x-1.85)/(-0.05)}-e^{-(y-3.15)/(-0.14)}}$
M2	$z= 1.96-1.03(0.014/((4(x-1.06)^2+0.05)(4(y-1.38)^2+0.06))+8.84ln2e^{(-4ln2/0.05)(x-1.06)^2-(4ln(2)/0.07)(y-1.38)^2})$
M3	$z=1204.85-913.03x+1391.56y+170.58x^2+3.62y^2-463.20xy$
M4	$z=3139.14-3175.62x+1469.88y+706.62x^2-5.50y^2-481.38xy$

M1: Extreme Cum; M2: Voigt2DMod; M3: Poly2D and M4: Poly2D.

### Comparing model predictions to monitoring data 2019 and model applied to 2020

**Figure 9:**
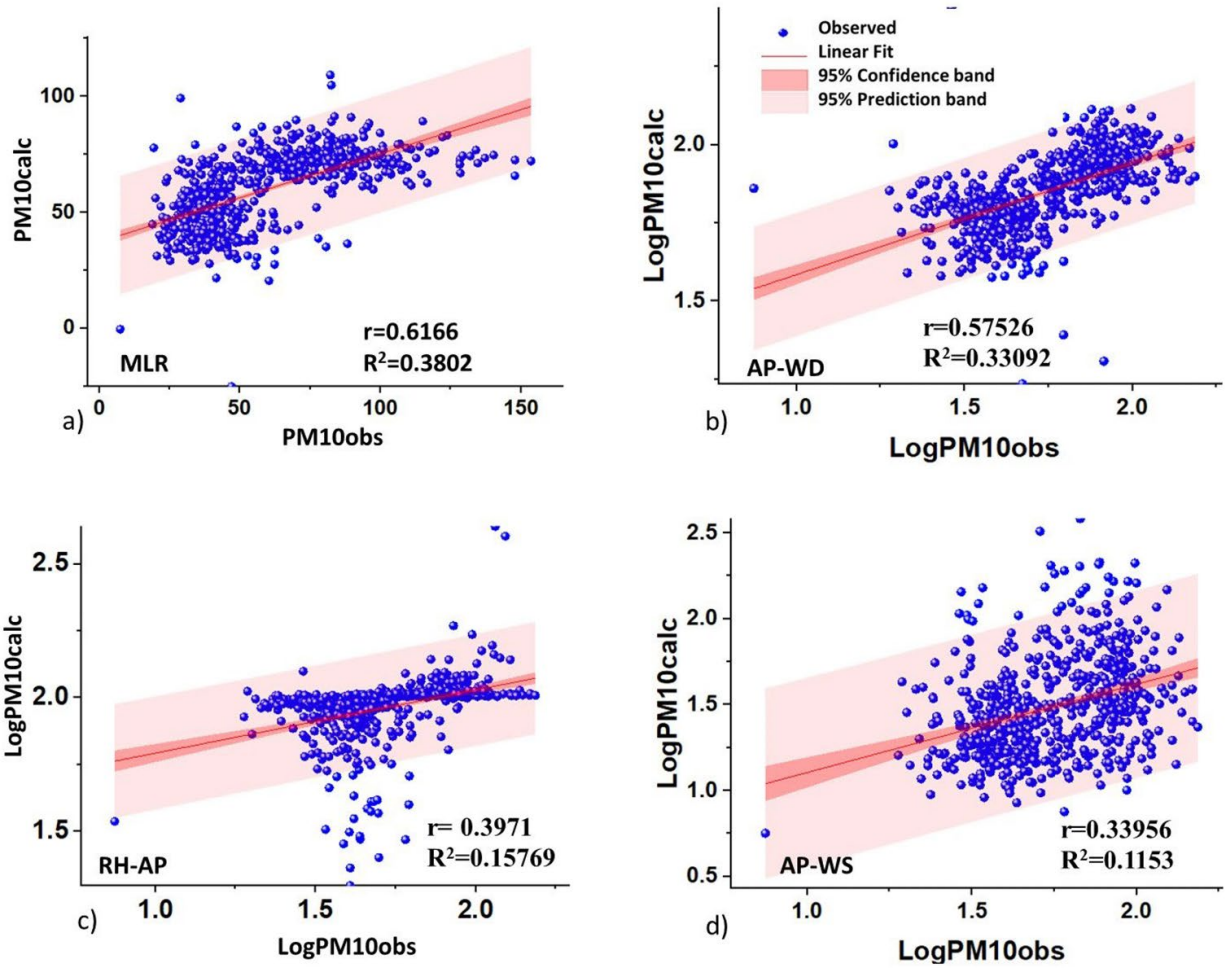
Calibrations 2019: (**a**) multiple linear regression, (**b**) Log AP- Log WS-Log PM$_{10}$ combinations, (**c**) Log RH-Log AP-Log PM$_{10}$, (**d**) Log AP-Log WS-Log PM$_{10}$.

**Figure 10:**
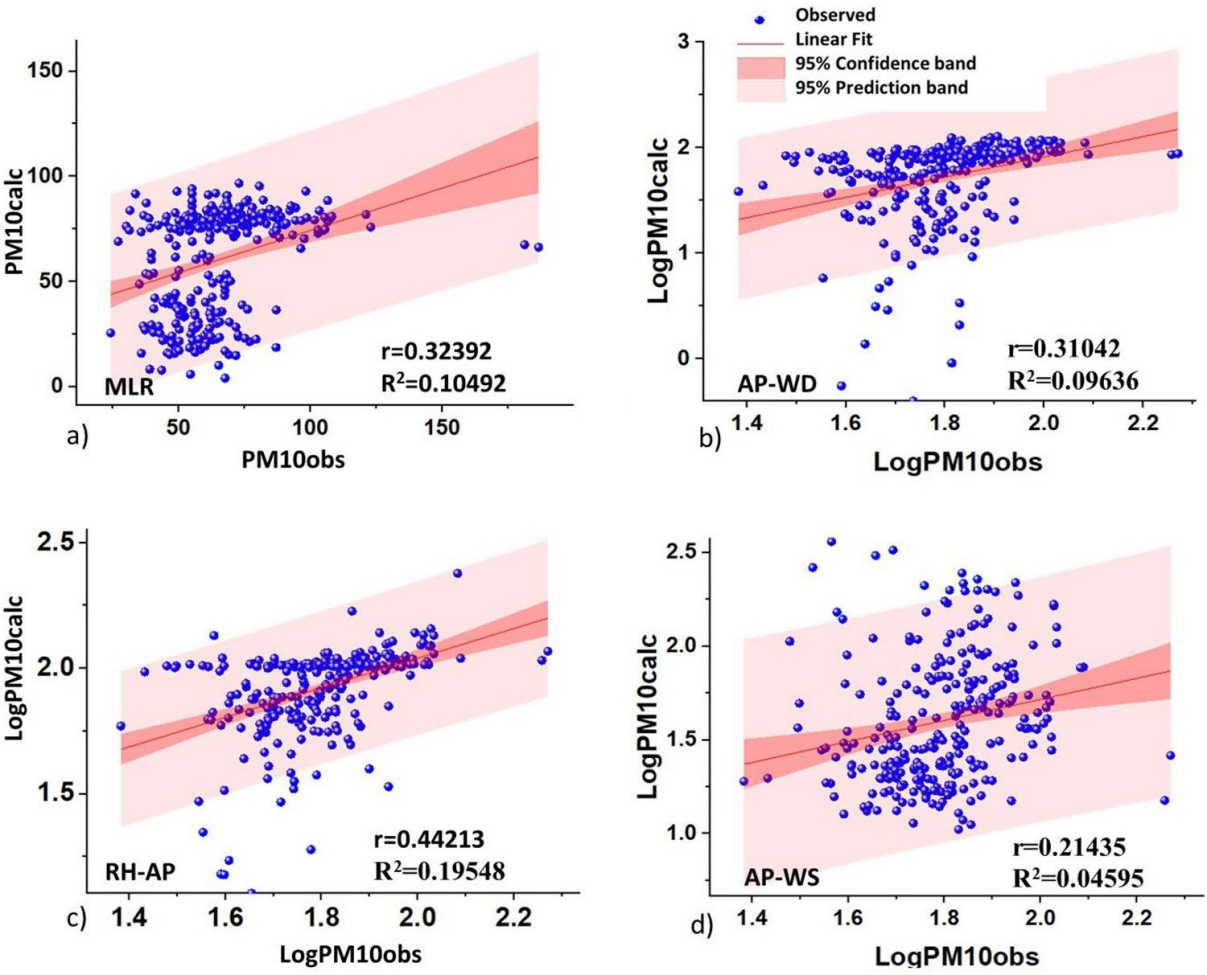
Evaluation 2020: (**a**) Multiple Linear Regression, (**b**) Log AP- Log WS-Log PM$_{10}$ combinations, (**c**) Log RH-Log AP-Log PM$_{10}$, (**d**) Log AP-Log WS-Log PM$_{10}$.

Figure 9 shows the data fit between the observed PM$_{10}$ and the calculated (modeled) PM$_{10}$ for the year 2019. While, Fig. 10, represents the data fit in the 2020 assessment. The calibration of the models evaluated by the correlation coefficient indicates that the MLR performed better than the other models developed $(\text{r}=0.6166)$ between the outputs (modeled PM$_{10}$) and the observed data (Fig. 9a). Also, the three-dimensional function that combined the LogAP-LogWD-LogPM$_{10}$ (Fig. 9b) presented a moderate correlation $(\text{r}=0.5753)$ unlike the other two 3D models. However, Table 5 shows that the RMSE for the MLR was higher (RMSE= 12.9226) relative to the others, but comparable to the modeling errors of another study $(\text{RMSE}=10.64-26.08, \,\text{T}-\text{AP}-\text{RH}-\text{WS})$^45^. The 3D models had smaller errors ($\text{RMSE}=0.0989$ a 0.2776) because the algorithm for 3D models by regression fitting generates more complex interactions between input and output data. The NSE criterion for MLR (NSE=0.3804) was closer to unity, and was also comparable to Nguyen’s fitting errors^45^ (between 0.26 and 0.53 ).

**Table 5:** Statistical comparison results between the models studied for PM$_{10}$. Model 1: Model 3D (LogAP-LogWD-LogPM$_{10}$); Model 3D (LogRH-LogAP-LogPM$_{10}$); Model 3D (LogAP-LogWS-LogPM$_{10}$); CAL: Calibration and EVA: Evaluation.

Metrics								
Model	Pearson’r		R$^2$		RMSE		NSE	
	CAL	EVA	CAL	EVA	CAL	EVA	CAL	EVA
MLR	0.6166	0.3239	0.3802	0.1049	12.9226	23.9983	0.3804	− 2.7214
Model 1	0.5753	0.3019	0.3309	0.0911	0.0989	0.3773	0.0544	− 8.0018
Model 2	0.3971	0.4435	0.1577	0.1967	0.1065	0.1545	− 1.2841	− 1.5304
Model 3	0.3396	0.2144	0.1153	0.046	0.2776	0.3305	− 2.9502	− 9.0345

The evaluation of the models applied to the 2020 data showed a decrease in correlations, with the exception of the combined 3D model of RH-AP-PM$_{10}$ and MLR which showed slightly higher correlations with $(\text{r}=0.4435)$ and $(\text{r}=0.3239)$ respectively. The RMSE for the MLR doubled relative to its calibration ($\text{RMSE}=23.9983$) and the values of the NSE criterion were all negative, but the NSE of the MLR and the 3D combined AP-WS models were closer to unity error, with values of − 2.7214 and − 1.5304, respectively. Consequently, two relevant aspects were highlighted:

- Conditions in 2019 were characterized by intense anthropogenic activity. While in 2020, the cessation of activities at the beginning of the pandemic showed changes during the months of blocking and subsequent reactivation (May). These changes were reflected in the wind patterns, especially for SS, which presented a dominant direction towards the south in 2019. While in 2020 it was from east to south east, associated with the increase in PM$_{10}$.
- The results should be considered significant for predicting PM$_{10}$ concentration. However, it is believed that the inclusion of new predictor variables related to TI base altitude, aerosol re-suspension, vehicular traffic and discriminations of anthropogenic and geogenic sources^57^ would help to improve the model to compare with others in order to minimize human health risks in times of pandemic.

### Comparison of research on atmospheric quality in the city of Lima, Peru

The results of research conducted by different authors on air quality related to PM$_{10}$ in the city of Lima were compared with the present study.

- This research provides an easy and practical method with effective and reliable results through the development of statistical prediction models for PM$_{10}$, based on multiple linear regression, use of three-dimensional logarithms and principal component analysis, under the influence of meteorological variables in the warm and cool season in southern Lima. This technique allows testing the applicability of the models and reveals the spatial distribution dynamics of PM$_{10}$, strengthening decision making in environmental management related to the protection of human health through the prevention and control of PM$_{10}$ air pollution in the context of constant urban growth.
- Silva et al.^58^ evaluated the PM$_{10}$ pollutant in the city of Lima over a 6-year period (2010-2015), showing that the highest PM$_{10}$ concentrations were observed in the eastern part of the city, mainly in the summer (December to March). In addition, the authors identified large open spaces, vehicular traffic and the commercialization of rubble, bricks and cement as the main sources of particulate matter. These results are similar to those reported in this research; however, the authors conducted the research in a period before the COVID-19 pandemic, which reflects a stable situation in environmental conditions.
- Reátegui-Romero at al.^59^ conducted a study on PM$_{10}$ and PM$_{2.5}$ pollutants during 2 months (February and July 2016), showing that the highest PM$_{10}$ concentrations were observed in the northern area of Lima, and relative humidity is inversely proportional to PM$_{10}$ concentrations, with higher peaks observed in the summer month (February). The authors’ results coincide with the findings of this research for such months, which are explained through the seasonal behavior pattern of South Lima and in the 3D model that demonstrates the influence of association between RH and AP on PM$_{10}$ (Fig. 8a).
- Sanchez et al.^12^ used the WRF-Chem to predict PM$_{10}$ concentrations in Lima during April 2016, showing that there is a higher PM$_{10}$ concentration in areas with greater impact of vehicular traffic, reaching values of 476.8 $\upmu$g/m$^{3}$ for the *Santa Anita* station. The authors related temperature, relative humidity and wind speed, in addition to the incorporation of topographic and meteorological data that increased the accuracy in terms of normalized mean bias for PM$_{10}$ based on the emissions inventory. In contrast, this research was based on the modeling of air quality through the exclusive relation of meteorological variables with PM$_{10}$, showing the limitations of the applied models that could be enhanced with the inclusion of geomorphological factors, among others.
- Cordova et al.^34^ evaluated the PM$_{10}$ pollutant in Metropolitan Lima during 2017 and 2018, mentioning that the main sources were the vehicle fleet, the industrial park and overpopulation, reaching maximum values (974 $\upmu$g/m$^{3}$) at the *Huachipa* station for the summer months (December–March). Artificial neural networks, specifically, the Long Short-Term Memory model under two validation schemes were used to predict PM$_{10}$ concentrations. The results showed good prediction performance for both low concentrations and critical episodes. The model presented a potential application for South Lima and could be compared with the simple methodology (MLR, 3D and PCA) applied in this research. However, an analysis of the RMSE errors calculated in both applications resulted in discrepant values (statistical methods: 0.0989 to 23.9983; ANN: 10.573 to 64.297) and very close Spearman correlations (MLR: 0.6166; 3D RH-AP Model: 0.5753; ANN: 0.517–0.756).

## Limitations

This study has some limitations. There are gaps, about 67% are valid data (2019–2020). It is also limited to the application of statistical functions such as MRL, PCA and logarithmic functions using the factors, coefficient of determination, correlation coefficient, RMSE and NSE. The model is proposed with data from 2019 and extrapolated to the following year due to the limited availability of data in 2020. The application of three-dimensional models is limited by their low R$^2$ (for the year 2020). The number of data represents a relatively short period (two years). A more extended period of hourly data may have allowed a more rigorous statistical analysis and more conclusive results.

## Conclusions

A statistical modeling approach has been applied to predict PM$_{10}$ concentration in two locations in South Lima (SJM and SS) both before (2019) and during the COVID-19 pandemic (2020), as a function of meteorological variables. The PCA evidenced the seasonal influence explained in the various combinations of meteorological variables on the distribution of PM$_{10}$. The SJM district presented moderate to poor PM$_{10}$ quality levels versus most acceptable values in SS. Calibration of the statistical models in 2019 demonstrated a better (significant) fit for the multiple linear regression model than the 3D modeling, while evaluation of the models in 2020 generated lower determination factors. Thus, this research strengthens the application of statistical models in predicting the spatial distribution of PM$_{10}$, providing scientific support in decision making related to public health protection. As future work, we consider developing new models under the machine learning approach through the application of geostatistical models to compare the accuracy in the prediction of air quality for PM$_{10}$. Likewise, address the analysis of air pollution before, during and after the pandemic using diagnostic measures for the class of nonparametric regression models with symmetric random errors, which includes all continuous and symmetric distributions^60^.

## Data Availability

The collection and statistical processing of the data was carried out under the authorization of *Dirección General de Salud Ambiental*, the governmental entity responsible for the sanitary surveillance of air quality and meteorology. The datasets are available in the repository, http://www.digesa.minsa.gob.pe/DEPA/aire_lc/lima_callao.asp.
